# Repurposing Gaucher disease therapy for Saposin C deficiency: Proof-of-concept with eliglustat

**DOI:** 10.1016/j.ymgme.2026.109865

**Published:** 2026-06

**Authors:** Carmen Minea, Patrick B. Deegan

**Affiliations:** Lysosomal Disorders Unit, Cambridge University Hospitals Hills Rd, Cambridge CB2 0QQ, UK

**Keywords:** Saposin C deficiency, Substrate reduction therapy, Gaucher disease, Search terms, Prosaposin, Saposin c deficiency, Gaucher Disease

## Abstract

Saposin C (Sap C) deficiency (GDSAPC, OMIM #610539, ORPHA:309252) is an ultra-rare autosomal recessive disorder caused by mutations in the *PSAP* gene. Sap C functions as an essential activating cofactor of glucosylceramidase (GCase) and facilitates the degradation of glucosylceramide in the lysosome. In the absence of its activator, GCase is structurally intact but its function is impaired, leading to pathological accumulation of glucosylceramide in the lysosome.

Sap C deficiency resembles another rare autosomal recessive disorder, Gaucher disease (GD), in which pathogenic variants in the *GBA1* gene result in GCase deficiency and accumulation of glucosylceramide within the lysosomal compartment of the mononuclear phagocyte system. Despite the overlapping clinical features of Sap C and GD, the therapeutic implication is utterly different.

To date, no specific therapy is approved for Sap C deficiency. Enzyme replacement therapy (ERT), highly effective in GD is biologically implausible in Sap C deficiency, where the GCase structure is normal but lacks its essential activator for its functions.

In GD, substrate reduction therapy (SRT) is an alternative to ERT by limiting the synthesis of glucosylceramide through inhibition of glucosylceramide synthase. Given that substrate accumulation is the common pathological consequence in both GD and Sap C deficiency, we hypothesized that SRT with Eliglustat, a potent glucosylceramide synthase inhibitor, could also benefit patients with Sap C deficiency.

We describe the first documented therapeutic attempt using eliglustat in Sap C deficiency. A 47-year-old patient with *PSAP* mutations causing Sap C deficiency who presented with features similar to those seen in GD, has received Eliglustat over the course of 9 years, demonstrating an improvement in her hepatosplenomegaly, haematological parameters, biomarkers and bone density, providing proof-of-concept that Eliglustat can be of benefit when the GCase cofactor is deficient.

However, no improvement was observed in the patient's seizure activity where future brain penetrant molecules may be of benefit.

## 1 Introduction

Saposin C (Sap C) deficiency (GDSAPC, OMIM #610539, ORPHA:309252) is a rare autosomal recessive disorder caused by mutations in the *PSAP* gene. Prosaposin is the precursor of four homologous proteins: saposins A, B, C, and D [1]. Sap C deficiency presents with clinical features similar to those observed in Gaucher disease (GD) [1].

Each of the four saposins serves as an essential activator for specific lysosomal hydrolases involved in glycosphingolipid degradation in the cells\ [2]. Sap C is specifically required for the proper functioning of glucosylceramidase (GCase), facilitating in vivo the degradation of glucosylceramide within the lysosome [1].

Although mechanism of interaction between of Sap C and GCase interaction remains unclear, proposed a theories involve a) solubilizer model, in which Sap C extracts the lipid from the lysosomal membrane in a soluble form and presents it to GCase for hydrolysis, and b) liftase model, in which Sap C is attached to the membrane helping with the exposure of the lipid to the GCase. [3]

The coordinated integrity of both Sap C and the GCase is necessary for the degradation in vivo of glucosylceramide within the lysosome.

In Gaucher disease (GD, OMIM #230800, ORPHA355), another rare autosomal recessive disease, variants in the *GBA1* gene result in a deficiency of GCase, causing the build-up of glucosylceramide in the lysosome.

The lack of GCase activity affects mainly cells of the mononuclear phagocyte system as they are responsible for breaking down cell membranes rich in sphingolipids in the process of phagocytosis. Tissue infiltration by these cells is responsible for the common manifestation of the disease which includes hepatosplenomegaly, cytopenia, monoclonal gammopathies, bone disease, including osteoporosis, and neurological manifestation. [4]. Based on the presence of neurological features, several attempts have been made to classify the different forms of Gaucher disease (GD). A hallmark of the so-called neuronopathic forms of GD is impaired saccadic eye movements [5]. The non-neuronopathic form is classified as Type 1 GD, lacks the saccadic movement abnormality and other features like cerebellar ataxia and myoclonus. Nevertheless, an increased risk of Parkinson's disease is observed and peripheral neuropathy has also been suggested as a neurological manifestation [6], [7]. The neuronopathic forms are categorized as Types 2 and 3. Patients with Type 2 GD typically die within the first few years of life [8], while Type 3 GD have a chronic and progressive form of the disease [5].

The clinical features observed in patients with Sap C deficiency may overlap with those seen in Gaucher disease (GD) and is not surprising that recent publications have referred to Sap C deficiency as an Atypical Gaucher Disease or Gaucher-Like Disease [9] [10] Manifestations such as hepatosplenomegaly and cytopenia have been consistently reported in the seventeen published cases of Sap C deficiency to date. In contrast, neurological features have been reported inconsistently; however, when present, they typically include seizures. Ophthalmoplegia has been described in only one patient. (See Table 1).

**Table 1:** List of previously published patients with Sap C deficiency. The features are as reported at the time of publication.

Number	Ethnicity	Consanguinity	Sex	Age of Presentation	Genotype	Systemic manifestations	Neurological manifestations	Treatment attempted	References
1	Swiss		Female	4	1. c.1145G > T (p.Cys382Phe) 2. N/A	Hepatosplenomegaly	Severe mental deterioration, seizures		[9], [22]
2	Spanish	No	Male	8	1. c.1144 T>G p.(Cys382Gly) 2.p.Q430X	Hepatosplenomegaly, no skeletal abnormalities, anaemia in one occasion	Seizures, severe ataxia, tremor, ophthalmoplegia, dysarthria, and spastic tetra paresis		. [29], [30], [31]
3	Chinese	No	Male	2	1. c.1133C > G(p.Pro378Arg) 2.delE2-E7	Hepatosplenomegaly, epistaxis, thrombocytopenia and mild anaemia, bone involvement	No neurological manifestation at age 10 and 4 months		[32]
4	French		Female	7	1. c.943 T > A (p. Cys315Ser) 2. c.1 A > G** (p.M1V)	Splenomegaly, leukopenia, thrombocytopenia	Generalized epileptic crisis, intellectual decline, dysmetria, myoclonic jerks, no ophthalmoplegia by the age 16	Miglustat 12 months duration with slightly improvement in her neuropsychological state but persistent poor life quality	[21]
5	Indian Sikh		Female	5	1. c.1024_1044del (p. Phe342_Lys348del) 2. c.1024_1044del (p. Phe342_Lys348del)	Hepatosplenomegaly	No neurological manifestation at age 5		[1], [9]
6		Yes	Female	12	1. c.1069 T > C (p. Cys357Arg) 2. c.1069 T > C (p. Cys357Arg)	Splenomegaly, thrombocytopenia	Myoclonic jerks, generalized seizures,		[33]
7	Emirati	Yes (siblings: 8)	Male	7	1.c.1005 + 1G > A (Premature stop codon at Sap C) 2.c.1005 + 1G > A (Premature stop codon at Sap C)	Cholelithiasis, hepatosplenomegaly, growth retardation, anaemia, thrombocytopenia, osteopenia	Intractable seizure, attention deficit disorder	Miglustat used on a trial bases, decreased level of lyso-Gb1 noticed but poor compliance due to side effect	[10]
8	Emirati	Yes (sibilings:7)	Male	2	1.c.1005 + 1G > A (Premature stop codon at Sap C) 2.c.1005 + 1G > A (Premature stop codon at Sap C)	Growth retardation, hepatosplenomegaly, growth retardation, poor appetite	No neurological manifestation at age 10		[10]
9	Emirati	Yes (siblings: 10)	Female	5	1c.1005 + 1G > A (Premature stop codon at Sap C) 2.c.1005 + 1G > A (Premature stop codon at Sap C)	Hepatosplenomegaly, thrombocytopenia, cholelithiasis, constipation, chronic fatigue (Vit D deficiency), autoimmune hypothyroidism	Refractory myoclonic epilepsy		[10]
10	Emirati	Yes (siblings: 9)	Female	23	1c.1005 + 1G > A (Premature stop codon at Sap C) 2.c.1005 + 1G > A (Premature stop codon at Sap C)	Anaemia, hepatosplenomegaly	No neurological manifestation at age 23		[10]
11	Pakistan	Yes	Female	12	1.c.1076 A > C p. (Glu359Ala) 2.c.1076 A > C p. (Glu359Ala)	Hepatosplenomegaly, thrombocytopenia, kyphosis, myopia	Vestibular dysfunction, hearing impairment		[9]
12	Pakistan	Yes	Female	14	1.c.1076 A > C p. (Glu359Ala) 2.c.1076 A > C p. (Glu359Ala)	Hepatosplenomegaly, thrombocytopenia, kyphosis	Vestibular dysfunction, hearing impairment		[9]
13	Pakistan	Yes	Male	20	1.c.1076 A > C p. (Glu359Ala) 2.c.1076 A > C p. (Glu359Ala)	Hepatosplenomegaly, thrombocytopenia, kyphosis, myopia	Vestibular dysfunction, hearing impairment		[9]
14	Pakistan	Yes	Female	19	1.c.1076 A > C p. (Glu359Ala) 2.c.1076 A > C p. (Glu359Ala)	Hepatosplenomegaly, thrombocytopenia, kyphosis	Vestibular dysfunction (tandem gait and Romberg test impaired) hearing impairment		[9]
15	India	Yes	Male	1 month	1. c. G1228T (p. Glu410ter) 2. c. G1228T (p. Glu410ter)	Anaemia, thrombocytopenia, poor feeding, moderate hepatosplenomegaly, fundus examination shows bilateral cherry red spots, bronchopneumonia	Tonic–clonic seizures, hypotonia of all limbs, sluggish deep tendon reflexes and finally encephalopathic		[34]
16	Polish (MZ)	No (sibilings:17)	Male	2	1.C.1 A > T (pMet1Leu) 2. C.1046 T > C (p. Leu349Pro)	Hepatosplenomegaly, osteopenia, cachexia, bone remodelling, anaemia and severe thrombocytopaenia.	Peripheral nervous system involvement, (documented only after Miglustat therapy)	Miglustat for 2 years with no improvements on his hepatosplenomegaly, haematological parameters or glucocerebrosidase serum level.	[11], [14]
17	Polish (AZ)	No (sibilings:16)	Female	29	1.C.1 A > T (pMet1Leu) 2. C.1046 T > C (p. Leu349Pro)	Hepatosplenomegaly, anaemia, thrombocytopaenia,	Mild peripheral sensory-motor axonal neuropathy; jerks		[11], [14]

In Sap C deficiency, the enzymatic activity of glucocerebrosidase (GCase) measured in vitro is typically normal, because the standard assay uses sodium taurocholate, a bile salt that directly activates GCase and bypasses the physiological requirement for Sap C [11], [12]. In contrast to Gaucher disease, where reduced GCase activity is a primary diagnostic marker, Sap C deficiency does not result in decreased enzyme activity in routine laboratory testing [11]. Therefore, in patients presenting with clinical features suggestive of Gaucher disease but demonstrating normal GCase activity on enzymatic assay, Sap C deficiency should be considered [11].

As expected, elevation of CCL18/PARC and chitotriosidase, known biomarkers in GD, has also been observed in patients with Sap C deficiency [13], [14].

To date, no recognized treatment is available for Sap C deficiency. However, Gaucher disease, the disorder most closely resembling Sap C deficiency, has benefited from well-established treatment strategies. The standard therapy for classical Gaucher disease has been enzyme replacement therapy, which has been used in the management of GD for over three decades. The enzyme replacement therapy aims to deliver exogenous enzyme to the lysosomes in the macrophage, by display of terminal mannose residues on the glycan component of the glycoprotein to engage with macrophage mannose receptors and allow for the breakdown of storage material [15]. An alternative therapeutic strategy involves reducing the endogenous production of glucosylceramide, the accumulated substrate, by inhibiting glucosylceramide synthase [16]. This mechanism of action is shared by miglustat and eliglustat, both approved for clinical use in GD. Due to its limited efficacy and tolerability, miglustat is generally considered a second-line therapy for patients with mild to moderate Type 1 Gaucher disease, particularly when enzyme replacement therapy (ERT) is not an option [17] [18].In contrast, eliglustat has shown significant clinical benefits, including improvements in hepatosplenomegaly, bone disease, and haematological parameters. It is now used as an oral alternative for patients with Type 1 Gaucher disease [19], [20].

In patients with Sap C deficiency, where GCase is functionally intact but lacks its activator, successful treatment with exogenous enzyme therapy is implausible. Substrate reduction therapy can offer a different therapeutic approach by addressing the production of glucosylceramide.

In the seventeen of the Sap C reported patients, treatment was attempted in three of them with miglustat as a substrate reduction therapy with no consistent benefit. These patients genotype, phenotypes and response to treatment are presented in Table 1.

Here we present the case of a patient with Sap C deficiency where Eliglustat was used with good effect on the systemic manifestation of the disease.

## 2 Clinical case

AZ is a 48-year-old female who first presented at the age of 29 with hepatosplenomegaly, anaemia and thrombocytopenia. A diagnosis of Gaucher disease was the first suggestion due to her brother's (MZ) known disease who presented symptoms at the age of two. Our patient and her brother were previously described by Tylki-Szymanska et al. in 2007 and 2011 [11], [14].They both were found to have two mutations in the *PSAP* gene and, as expected, their GCase activity tested in leucocytes and fibroblasts was normal [11].

Having migrated to the UK in 2011, AZ presented to our service whilst pregnant with a very low platelet count of 58 × 10^9/^L. Despite the low platelet number, no easy bruising or bleeding was reported.

To offer support during pregnancy, substrate reduction therapy with Miglustat was considered but rejected due to the pregnancy safety concerns. The same applied to the possibility of Eliglustat which was at that time in clinical development. AZ returned to Poland where she successfully delivered a healthy boy via caesarean section with platelet support.

Shortly after delivery, she began to experience myoclonic jerks affecting all limbs, which became progressively more frequent.

Five years later she returned to our service in the UK. Later, in the same year she presented her first generalized seizure; levetiracetam was initiated by her neurologist.

**Fig. 1:**
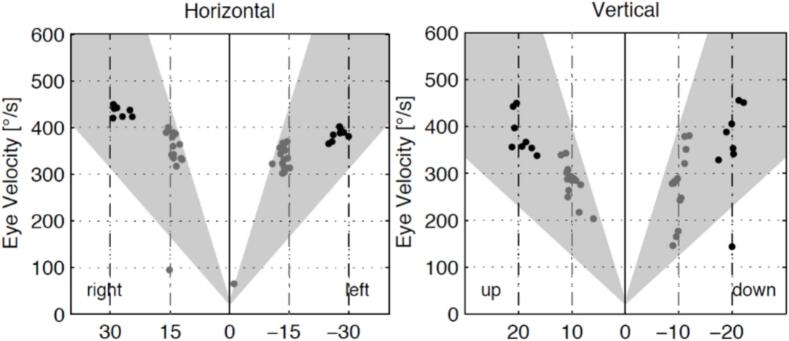
Video-oculography, demonstrating normal reflexive horizontal (left) and vertical (right) saccadic eye movement velocity, by “EyeSeeCam” device, Munich Germany.

**Fig. 2:**
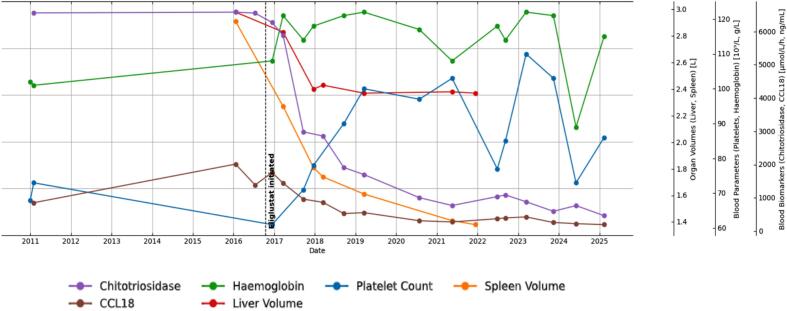
Trend of different parameters after the initiation of Eliglustat.

On examination, an enlarged liver and spleen was noted. Baseline blood count, organ volumes and biomarkers are shown in Fig. 2. Saccadic eye movements were normal, as determined by clinical examination and by saccadometry (EyeSeeCam) (Fig. 1).

Eliglustat was proposed to the patient in September 2016, and she accepted. Her CYP2D6 genotype predicted an extensive metaboliser status and consequently Eliglustat was initiate with good tolerance at 84 mg twice daily.

Subsequent monitoring has showed significant improvement in her biomarkers (PARC/CCL18 and chitotriosidase), platelet count and hemoglobin concentration (Fig. 2).

**Fig. 3:**
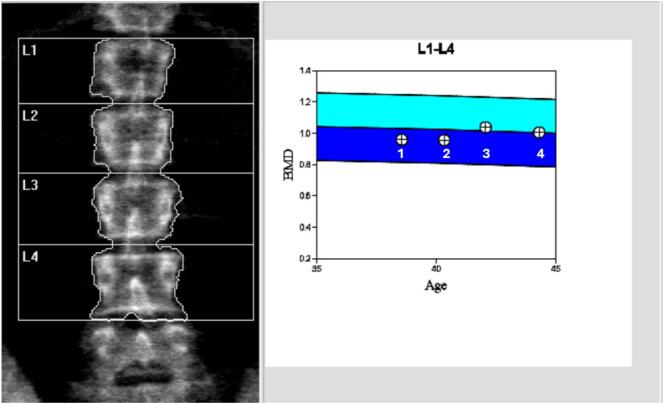
Bone mineral density (BMD) at the lumbar spine. Crossed circles show BMD (g/cm2) according to age (years), after the start of Eliglustat. *Z*-scores are as follows: 1: −0.6, 2: −0.6, 3: 0.2, 4: 0.

Volumetric analysis of liver and spleen carried out from serial Magnetic resonance imaging, showed a salutary effect of Eliglustat on her spleen and liver as shown in Fig. 2. Bone mineral density at the lumbar spine has increased (Fig. 3).

In 2019, a monoclonal band has been noticed and the regular monitoring of her paraprotein of 3 g/L showed no changes.

Despite improvement in systemic features and a reduction in fatigue, the patient continued to experience frequent myoclonic jerks and generalized epileptic seizures. Several adjustments to her antiepileptic regimen were made to control seizure frequency. Sodium valproate was initially introduced but later replaced with lamotrigine, which was used in combination with levetiracetam.

## 3 Discussion

To date, this is the first reported case in which treatment with eliglustat has been attempted in a patient with saposin C (Sap C) deficiency. Previously, miglustat has been used in patients with Sap C deficiency, with little to no effect on the disease [10], [14], [21]. One of these patients was the brother of our current patient [14]. He received miglustat at a dose of 300 mg daily; however, after two years of treatment, there was no improvement in hepatosplenomegaly, haematological parameters, or serum glucocerebrosidase levels [14]. In another case, twelve months of miglustat use in an adolescent with Sap C deficiency led to some improvement in neurological symptoms, but no effect was observed on her overall quality of life [21]. Mohamed et al. in 2022 reported the use of miglustat in a 14-year-old male with Sap C deficiency and neurological symptoms. Although there was a reduction of the substrate concentration, the patient's overall compliance was poor due to adverse effects [10]. In contrast, eliglustat treatment in our patient has led to a gradual improvement in systemic disease since 2016. After six years of treatment, spleen volume decreased by more than 50%, and liver volume was reduced by 18%. Unfortunately, a recent MRI could not be obtained due to the patient's distress with the procedure. Regarding her haematological markers, the platelet count improved over the course of treatment. Her hemoglobin levels fluctuated over the years, but this was attributed to an underlying iron deficiency anaemia secondary to gynaecological bleeding with intolerance to iron therapy. The response of our patient to eliglustat was comparable to that seen in patients with Gaucher disease type 1, in which the underlying genetic defect affects the GCase [19], [20]. During the first years on eliglustat, she reported an improvement in her fatigue, and her overall quality of life was significantly better according to recognized non-disease specific questionnaire. Despite this favourable systemic response, her myoclonic epilepsy remains poorly controlled.

AZ was initially considered to have a non-neuropathic form of GD- like disease due to Sap C deficiency [11]. As we have seen, the patient has developed myoclonic jerks [14] followed by generalized epileptic seizures later in life. The patient's brother had an early presentation with more significant systemic involvement without initial features suggesting neurological disease [11]. Subsequently, we learned from our patient that her brother died at 48 years of age.

It has been hypothesized that mutations which do not affect the cysteine residues responsible for forming the disulfide bridges, responsible for SAP-C structure and stability, may result in a phenotype resembling Type 1 Gaucher disease. In contrast, mutations that do affect these regions, or those that abolish the production of all saposins, may lead to a phenotype consistent with Type 3 Gaucher disease [1]. This hypothesis was based on cases published up to that time, including our patient and her brother, who were previously described as having the p.L349P mutation, which does not involve any of these structural regions [1], [11]. At the moment, this mutation has only been described in AZ and MZ. The second mutation identified in AZ and MZ is p.M1L is in the start codon of *PSAP* resulting in a null allele [11]. This latest mutation was reported in a heterozygous state in a newborn and his fetal sibling and this led to the abolition of prosaposin protein production [22].

From the seventeen patients published up to this date with SapC deficiency, six of them did not have neurological manifestations at the time of their first publication. These six patients include AZ and MZ. Since the initial publication by Tylki-Szymańska in 2007, AZ did develop myoclonus and subsequently generalized seizures later in life.

The phenotypic variability observed in Sap C patients, even among those sharing the same mutation, may be related to the duration of follow-up. This is seen in the case of AZ, in whom neurological manifestations appeared later in life. Alternatively, different phenotypes described in Sap C patients could be due to genetic modifiers; this was proposed by Klein et al., in a mouse model to be responsible for the phenotypic variability of Gaucher Disease [23]. The role that epigenetics may play on Gaucher disease severity was suggested by Biegstraaten et al. who described different diseases severity on monozygotic twins from consanguineous parents [24]. The onset of myoclonic jerks in our patient after her pregnancy and the clustering of her myoclonic seizures during her menstruation, raises the possibility of a hormonal role on both the appearance and the evolution of her disease.

Interestingly, Alaei et al. included Sap C deficiency patients within the classification of Gaucher as a neuropathic, non-conventional and atypical GD [25]. Abnormal saccadic eye movements are the defining features of Type 3 [5], whereas only one of 17 reported patients with Sap C deficiency had this feature. While the biology of Sap C is related to that of GD, it is not identical, and it is therefore not surprising that the neurological involvement in Sap C deficiency is atypical for GD. Another clinical entity, caused by deficiency of the lysosomal integral membrane protein type 2 (LIMP-2), which functions as a transporter for GCase into the lysosome, also results in the accumulation of storage material and elevated biomarkers, as seen in GD [26]. However, LIMP-2 deficiency manifests with neurological abnormalities and renal failure [26]. Like Sap C deficiency, LIMP-2 deficiency is genetically distinct from GD, being due to mutations in the SCARB2 gene [26]. Both Sap C and LIMP-2 deficiencies are rare compared to Gaucher disease, and the underlying pathogenic mechanisms and full clinical spectrum remain incompletely understood. Therefore, rather than approach them as subtypes of GD, it may be more appropriate to consider them as separate clinical entities with overlapping features.

Recognizing the similarities and differences between Sap C deficiency and Gaucher Disease has also therapeutic implication. As we previously mentioned, standard treatment with enzyme replacement therapy for GD may not be appropriate, but substrate reduction therapy with Eliglustat resulted in a clear response in the systemic manifestations of Sap C deficiency. Venglustat, a more potent glucosylceramide synthase inhibitor than eliglustat, crosses the blood-brain barrier effectively. Although still in clinical development, venglustat shows promise as a potential treatment for the neuronopathic manifestations of Type 3 Gaucher disease [27], [28]. It is still to be determined whether venglustat will be of benefit in Sap C deficiency.

## 4 Conclusion

To our knowledge, this is the first case where treatment with Eliglustat was attempted in Sap-C deficiency. As seen in our patient, substrate reduction therapy with Eliglustat offers an option to address the systemic aspect of the disease, but neurological manifestations remain unaddressed. Brain penetrant inhibitors of glucosylceramide synthase may offer an additional effect on the neurological manifestation of Sap C deficient patients.

## CRediT authorship contribution statement

**Carmen Minea:** Writing – review & editing, Writing – original draft, Data curation. **Patrick B. Deegan:** Writing – review & editing, Writing – original draft, Visualization, Supervision, Funding acquisition, Conceptualization.

## Declaration of competing interest

The authors declare the following financial interests/personal relationships which may be considered as potential competing interests: (Patrick Deegan is a consultant to Spur Therapeutics, Sanofi-Aventis and Takeda.Carmen Minea reports no conflicts of interest.)

## Data Availability

Data will be made available on request.
